# Design of Ti64/Ta Hybrid Materials by Powder Metallurgy Mimicking Bone Structure

**DOI:** 10.3390/ma16124372

**Published:** 2023-06-14

**Authors:** Francisco Alvarado-Hernández, Elena Mihalcea, Omar Jimenez, Rogelio Macías, Luis Olmos, Enrique A. López-Baltazar, Santiago Guevara-Martinez, José Lemus-Ruiz

**Affiliations:** 1 Unidad Académica de Ingeniería I, Universidad Autónoma de Zacatecas, Zacatecas 98000, Mexico; ingenierofah@gmail.com (F.A.-H.); emihalcea@itmorelia.edu.com (E.M.); ealopezb@uaz.edu.mx (E.A.L.-B.); 2 CUCEI, Universidad de Guadalajara, Zapopan 45100, Mexico; santiago.guevara@academicos.udg.mx; 3 Tecnológico Nacional de México (IT Morelia), DEPI, Morelia 58120, Mexico; d16121679@morelia.tecnm.mx; 4 INICIT, Universidad Michoacana de San Nicolás de Hidalgo, Morelia 58060, Mexico; luis.olmos@umich.mx; 5 IIMM, Universidad Michoacana de San Nicolás de Hidalgo, Morelia 58060, Mexico; jlruiz@umich.mx

**Keywords:** sintering, biomedical materials, mechanical strength, permeability, computed tomography

## Abstract

This work reports on the fabrication of a novel two-layer material composed of a porous tantalum core and a dense Ti6Al4V (Ti64) shell by powder metallurgy. The porous core was obtained by mixing Ta particles and salt space-holders to create large pores, the green compact was obtained by pressing. The sintering behavior of the two-layer sample was studied by dilatometry. The interface bonding between the Ti64 and Ta layers was analyzed by SEM, and the pore characteristics were analyzed by computed microtomography. Images showed that two distinct layers were obtained with a bonding achieved by the solid-state diffusion of Ta particles into Ti64 during sintering. The formation of β-Ti and α′ martensitic phases confirmed the diffusion of Ta. The pore size distribution was in the size range of 80 to 500 µm, and a permeability value of 6 × 10^−10^ m^2^ was close to the trabecular bones one. The mechanical properties of the component were dominated mainly by the porous layer, and Young’s modulus of 16 GPa was in the range of bones. Additionally, the density of this material (6 g/cm^3^) was much lower than the one of pure Ta, which helps to reduce the weight for the desired applications. These results indicate that structurally hybridized materials, also known as composites, with specific property profiles can improve the response to osseointegration for bone implant applications.

## 1. Introduction

Human bone is a very important component which supports the human body; thus, the loss of a part of the bone or a whole bone reduces the quality of life. Fortunately, the bone is an organ that is being rehabilitated throughout the life of humans, generating new bone cells, which allows the bone to recover from fractures or small bone losses. Nowadays, the bone rehabilitation, including the replacement of bones, part of bones or missing teeth by implants and implant-supported prostheses, is routine for surgeons. Implants are fabricated from different biocompatible materials such as titanium (Ti) and its alloys, tantalum (Ta), CoCrMo and 316L stainless steel [1]. Such implants allow for partially solving the problem of the lack of bone. Nevertheless, they show some disadvantages; the first one is related to the mechanical properties, which are much higher than the ones of the human bones—for example, the elastic modulus of the metallic materials used for bone regeneration ranges from 100 to 200 GPa. These values are high compared to the ones of human bones that range from 0.4 GPa for trabecular bone to 17.9 GPa for cortical bone [2]. This induces the well-known stress shielding phenomenon, which weakens the bone close to the metallic implant, causing embrittlement of the bone and inhibiting the bone remodeling, which leads to the loosening of the implant [3,4,5]. To overcome this inconvenience, porous materials called “scaffolds” have been developed to reduce the mechanical strength of the implants. Additionally, porous materials contribute to the acceleration of the bone ingrowth because they allow for the passage of the corporal fluids that contain the nutrients and elements that promote the creation of new bone. Moreover, this may extend the lifetime of the implants inside the human body, as has been pointed out by other authors [6].

Therefore, in recent decades, the development of porous materials has attracted the attention of different researchers with the aim of developing different techniques to fabricate porous materials. The space holder technique was first used because it offers the possibility to control the pore features in function of the type of the pore formers used, their shape and their quantity. This technique has been used to produce highly porous materials, and different works have been devoted to studying the mechanical properties [7,8,9,10,11]. It was demonstrated that the mechanical strength of the porous materials can be diminished up to that of the human bones by controlling the pore volume fraction [12,13]. Notably, in a recent work, the major achievements with this technique are summarized by Rodriguez-Contreras et al. [14]. Recently, another widely used alternative for producing porous materials has increased in popularity, and it involves the additive manufacturing (AM). Notable results have been reported for the AM used to fabricate cellular materials that mimic the structure that composes the spongy bone by using highly complex geometric shapes for the pores [2,15,16,17,18,19]. Specifically, the typical tests of mechanical properties obtained by AM are in the range of values presented by the spongy bone, because the porosity reached with this technique is really high, around 90%. Besides a reduction in mechanical properties, the addition of pores also helps to improve the flow properties of the metallic implants, which allows for the distribution of the bone nutrients through them. For such a case, the permeability of porous materials, fabricated either by a space holder or AM, had been experimentally measured [20,21] and numerically determined, and the obtained values range from 2 × 10^−8^ m^2^ to 1 × 10^−13^ m^2^ [22,23]. 

Another example of designing materials for bone implants is the ones reporting on functionally graded porous structures [24,25]. Al Zoubi et al. [24] assessed the mechanical properties of femoral stems from numerical simulations of a human femur and concluded that 70% of porosity in the core was the best option to reduce the stress shielding. Ahmadi and Sadrnezhaad published one of the first research articles reporting on the fabrication by powder metallurgy of components with bone-like configurations, comprising highly porous cores of different diameters surrounded by compact shells of Ti6Al4V. They determined that the elastic modulus follows a linear behavior as a function of the porous core diameter [26].

Systematic investigations indicate the intention of the hard tissue implants to mimic the real bone microstructure, which is a complex one, and different properties have to be fulfilled to obtain an optimum bone implant. As was mentioned, the bone is a very complex organ that is continually remodeled throughout life. By definition, bone remodeling is a process where osteoclasts and osteoblasts work sequentially in the same Bone Remodeling Unit (BRU) [27,28]. Osteoblasts generate new bone tissue, while osteoclasts resorb mature bone to maintain regular bone metastasis. This process allows the bone to regenerate, but it depends on the size of the created defect. When the defect size is higher than a critical size, an implant acting as a bridge is required to facilitate the formation of new bone. For this purpose, it is important to have a material that allows for a high rate of osteoblasts over its surface. Although Ti64 allows for the osteoblast formation, the growth rate is low compared to the one of tantalum, as was demonstrated in [29,30]. So far, due to the physical characteristics and the difficulty of processing, as it has a high melting point of 3017 °C, powder metallurgy has been the most feasible route for fabricating porous Ta materials [30,31,32]. The values of the mechanical properties reported had been low enough to be compared with the human bones. It was also demonstrated that tantalum allows for a better adhesion of the apatite (new bone precursor) under simulated body fluids, which is attributed to the oxide layer (Ta_2_O_5_) formed at the surface [33]. This leads to a faster rate of bone ingrowth, which improves the fixation of tantalum implants.

Overall, although the tantalum seems to be a better material for bone implants applications, it also shows a few drawbacks, such as its high cost due to its rarity and the fact that it is a heavy metal; dense tantalum is around eight times heavier than human bones. To overcome such disadvantages, one solution can be the fabrication of materials that can limit the amount of tantalum in multi-material components with a tailored architecture, which may mimic the structure and the properties of the replaced bones. Thus, motivated by this solution, we report on the development of a novel material composed of a highly porous Ta core and a dense Ti64 shell, fabricated by Powder Metallurgy (PM), which can accomplish the different functionalities of the bones. The key objectives of this study are investigating the co-sintering and the microstructural and mechanical characteristics of this two-layer material.

## 2. Materials and Methods

### 2.1. Sample Fabrication

**Figure 1:**
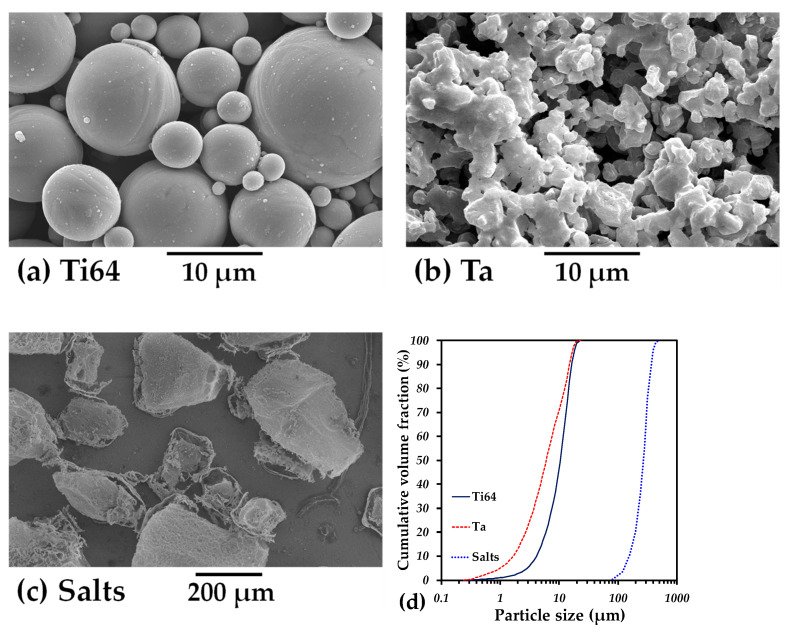
SEM images of initial powders: (**a**) Ti64; (**b**) Ta; (**c**) Salts; (**d**) particle size distribution of all powders.

A prealloyed Ti-6Al-4V powder with 0–25 µm spherical particles produced by Raymor, Boisbriand, QC, Canada (Figure 1a) and Ta powder with an irregular shape and the same particle size distribution (Figure 1b), furnished by Sigma Aldrich (St. Louis, MO, USA), were used. In addition, particles of ammonium bicarbonate ((NH_4_)HCO_3_) salt with an irregular shape and a size distribution between 100 and 500 µm (Figure 1c), furnished by Alfa Aesar, Ward Hill, MA, USA, were used as space holders to create large pores. 

The particle size distribution of all powders is shown in Figure 1d. First, we fabricated monolayer samples in order to obtain the mechanical properties of each component of the following two-layer material. Ti64 powder was manually mixed with a solution of polyvinyl alcohol (PVA) as a binder at 1 wt.% and then simply poured into an 8 mm diameter steel die and compacted at a pressure of 500 MPa. The PVA was subsequently removed by heating at 500 °C for a period of 30 min, and the furnace was purged under a high flow rate of Argon. Then, Ta particles and 50 vol.% salt particles were mixed in a TURBULA^®^ shaker-mixer (Muttenz, Switzerland) for 30 min at 34 rpm in dry conditions. Next, 1 wt%. of polyvinyl alcohol (PVA) was also manually added as a binder, and the mixture was poured into an 8 mm diameter stainless steel die and pressed at 500 MPa. Salt particles were eliminated in a furnace by heating at 180 °C for 6 h under argon atmosphere.

**Figure 2:**
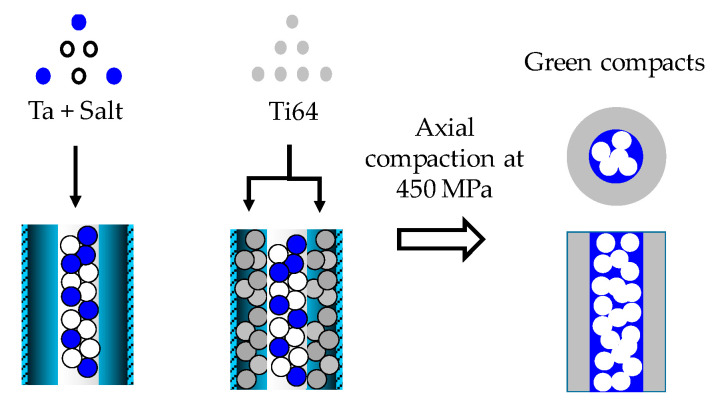
Schematic diagram illustrating the steps followed to fabricate the green compacts. Ti64 is in gray, Ta in blue and salt particles in white.

Second, two-layer samples were fabricated by combining dense Ti64 and porous Ta in a shell–core configuration. Two-layer green compacts were obtained as follows: first, a stainless steel tube with a 5.66 mm inner diameter was placed in the center of the 8 mm diameter stainless steel die. The inner diameter was chosen considering that we intended to have the same volume for the porous Ta core and Ti64 shell. The mixture of Ta and salt particles was poured into the tube, and a punch was used to slightly densify it and flatten the surface, as illustrated in Figure 2. The tube was extracted, and the outer space was filled with Ti64 powder (see [34] for the details of the process). Afterwards, the two-layer sample was pressed at 500 MPa. Next, the salt was removed, as explained above.

The sintering of all green compacts was carried out in a L75V vertical dilatometer (Linseis, Robbinsville, NJ, USA) at 1400 °C, with a heating rate of 25 °C/min and a plateau of 5 min in high-purity argon atmosphere. The dilatometer was purged to remove the air by flowing argon for 30 min before starting the heating. The temperature was determined based on the sintering temperature of Ti64 and Ta. As Ta has been sintered in the range of 1300 and 1500 °C [35,36], an intermediate temperature of 1400 °C was chosen to reduce the stresses generated during the densification of each layer in the two-layer sample.

### 2.2. Sample’s Characterization

The sintered samples were observed by a scanning electron microscope (SEM) after they were cut and surface-polished with SiC abrasive papers and alumina powders (until a 50 nm particle size), successively. The microstructure was observed with a Tescan MIRA 3 LMU (Tescan, Brno, Czech Republic) field emission scanning electron microscope (FE-SEM). The layer distribution and macroporosity analysis of the two-layer sample was achieved by 3D images that were acquired by computed microtomography (CMT) with a Zeiss Xradia 510 Versa 3D X-ray microscope (Zeiss, Jena, Germany). The beam intensity was 160 kV, which was enough to pass through Ti64/Ta samples with 7.4–7.5 mm diameters. The resulting voxel size was around 8 µm. The voxel size refers to a 3D measure analogous to the 2D pixel size, and it is related to both the pixel size and slice thickness. The voxel size is determined by the diameter of the sample, since the whole sample can be observed. In order to obtain quantitative data, the initial images were filtered, and then a thresholding operation was performed in order to obtain binary images with the phase of interest. In this case, three different phases were segmented: Ti64, Ta and pores. The image treatment for obtaining different information such as the pore size distribution, volume fraction, tortuosity, etc. is explained elsewhere [23]. The permeability of the porous layer was estimated by the numerical simulation of a fluid flow in the 3D real microstructure with the aid of Avizo^®^ software version 2019. Numerical simulations of the fluid flow were performed in the area of the core of the samples. Avizo^®^ solves the Navier–Stokes equations for a steady laminar flow of an incompressible Newtonian fluid. Then, the absolute permeability was calculated by using Darcy’s law; more details can be found elsewhere [37]. The boundary conditions used were a pressure differential of 30 kPa (130 kPa inlet and 100 kPa outlet), a fluid viscosity of 0.004 Pa s, which is the blood viscosity, and a convergence criterion of 1 × 10^−5^.

### 2.3. Mechanical Evaluation

To determine the mechanical strength of the samples, simple compression tests were performed on both kinds of samples: mono-material (Ti64 or porous Ta) and the two-layer sample with an Instron 1150 (Instron, Norwood, MA, USA) universal mechanical testing machine at a strain rate of 0.5 mm·min^−1^. The sample dimensions after sintering were in the range of 7.5–7.6 mm diameter and 11–12 mm height. The elastic modulus (E) and the yield strength (σ_y_) were estimated from the stress–strain curve, obtained from the load-displacement data provided by the machine.

## 3. Results and Discussion

### 3.1. Sintering Analysis

**Figure 3:**
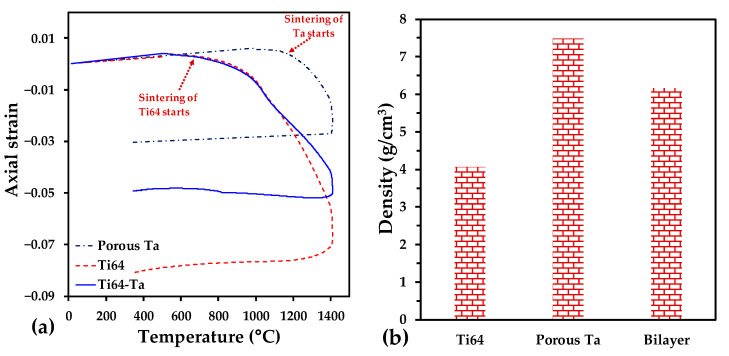
(**a**) Axial strain as a function of time and temperature during the sintering of the Ti64, porous Ta and two-layer sample; (**b**) density of samples after sintering.

The axial strain during the whole sintering cycle was determined by the instantaneous axial displacement obtained from the dilatometer data divided by the initial height of the samples. The variation in the axial strain during the sintering of the mono-material and two-material samples is plotted as a function of temperature in Figure 3a. The materials show, at first, a dilation due to their thermal expansion. Then, a shrinkage is observed, indicating that sintering is activated. It happens at 680 °C for Ti64 and at 1150 °C for porous Ta. The two-layer sample starts sintering at the same temperature as the Ti64 sample. A continuous shrinkage is next obtained for all samples. Finally, a quasi-linear shrinkage is observed during cooling. A slowing-down of the two-layer shrinkage can be noted at the temperature where the sintering of Ta is activated. It should be due to the constraint applied by the Ta layer on the Ti64 one. After sintering, the axial strain values of the two-layer sample are intermediate between those of the mono-material samples.

The weight density of the samples after sintering is presented in Figure 3b. The density of the Ti64 layer is 4.06 g/cm^3^, corresponding to a relative density of 0.92; meanwhile, that of the Ta layer is 7.5 g/cm^3^, corresponding to a relative density of 0.45. This value is close to the one reported for Ta scaffolds fabricated with 50 vol.% of NaCl [36]. The density of the two-layer sample is 6.16 g/cm^3^, which reduces the density of porous Ta by 20%. Note that the density of human bones is in the range 0.8–1 g/cm^3^ for trabecular bones and 1.2–2 g/cm^3^ for compact bones [38,39], and it has been suggested that the optimal weight density for an implant is 1.8 g/cm^3^ [40].

### 3.2. Microstructural Characterization

**Figure 4:**
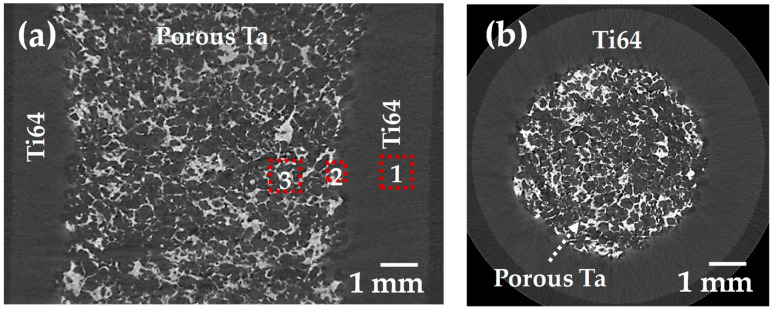
Virtual 2D slices of the transversal (**a**) and perpendicular (**b**) sections of the two-layer sample. The numbers 1–3 are the observed areas by SEM in Figure 7.

With the aim of detecting cracks in the two-layer sample after sintering, which might be caused by the difference in the densification kinetics of each layer, 2D virtual slices extracted from 3D images are analyzed (Figure 4). As can be observed, no crack is detected, and the resulting interface bonding is optimal in both radial and transversal sections. The large pores located at the interface between the two layers were likely created by the pore formers. Figure 4 also shows that the porous layer is correctly centered in the sample. The distribution of the Ta can be identified by a light gray in the image, and the Ti64 appears in dark gray, as the difference in density between Ta and Ti64 is around four times. Meanwhile, the pores appear black, and in this case, it was possible to segment the three phases from the images to obtain quantitative data.

**Figure 5:**
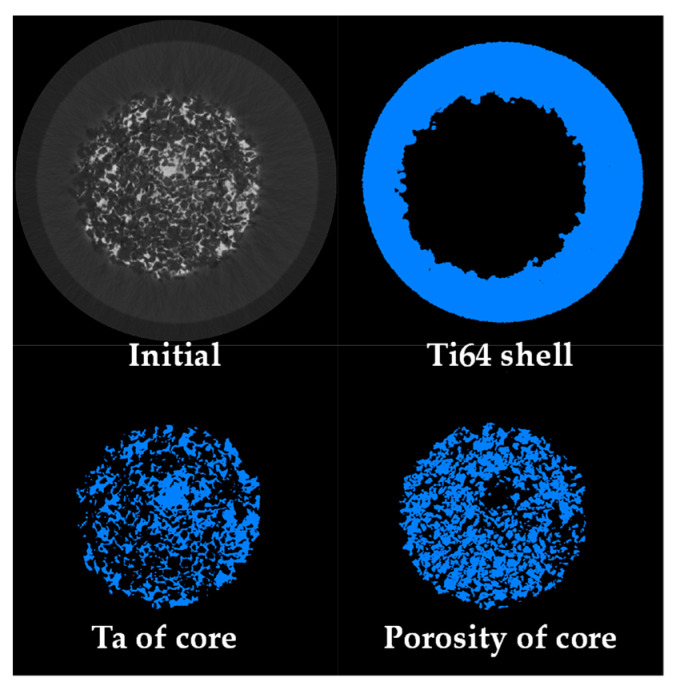
2D virtual slices of the two-layer sample, illustrating the segmentation of each phase of the sample.

**Figure 6:**
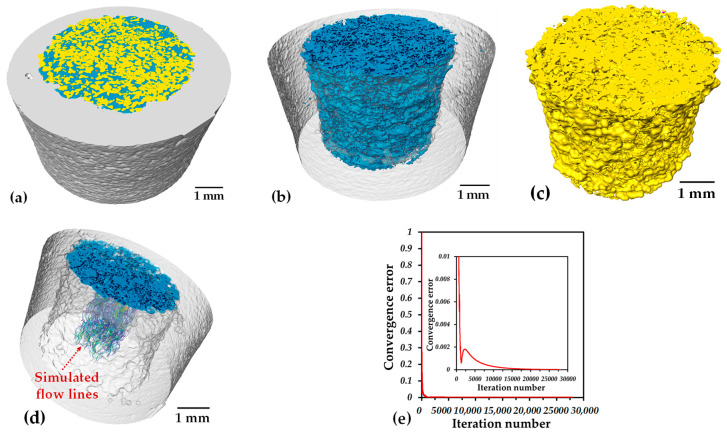
3D rendering of the two-layer sample: (**a**) Ti64, Ta and pores; (**b**) Ti64 and Ta; (**c**) pores; (**d**) simulated flow lines inside the porous core and (**e**) converge error plot.

The segmentation process was performed by selecting the gray levels that correspond to each phase as a function of the absorption of the X-ray. Once the gray level is selected, the image is transformed into a binary one. In the binary image, the phase of interest has a given intensity level of 1, and the rest of the image has one of 0. The range of the gray level intensity was divided into three ranges: low for pores, medium for Ti64 and high for Ta. The resulting binary images are shown in Figure 5. For illustrating the segmentation process, 2D virtual slices are shown in Figure 5; however, the whole 3D volume rendering is shown in Figure 6a, in which the three phases are clearly distinguished. It is also possible to confirm that no cracks developed at the interface and that the core and shell are clearly separated (Figure 6b). The porosity is shown in Figure 6c, and in addition, it was determined that the pore connectivity is 99%, which allows for the distribution of liquids inside the sample. The volume fraction of pores measured in the core is 55%. This value is in the same range as the one reported by Wu et al. [41], who improved the in vivo bone ingrowth in comparison to fully dense Ti6Al4V materials fabricated by AM. On the contrary, the porosity of Ti64 is too small to be determined from the 3D images because of the relatively high voxel size (8 µm). Thus, in order to have the volume fraction of pores for the whole sample it was assumed that the porosity of the shell is similar to the one of the monolayer Ti64 sample, sintered at the same conditions, which is 8%. Therefore, as the volume of the core is equal to the volume of the shell, the pore volume fraction on the whole sample can be estimated as 29%. 

**Figure 7:**
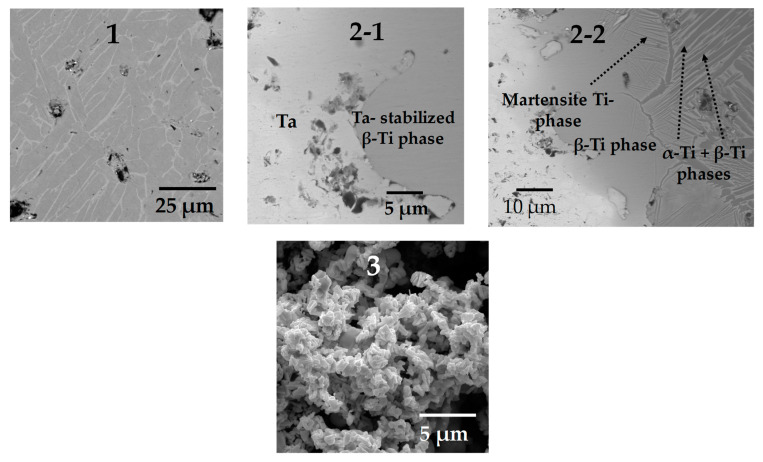
SEM images of the polished surface at the positions indicated in Figure 3a. (**1**) shell of Ti64, (**2-1**,**2-2**) interface between Ta core and Ti64 shell and (**3**) Porous Ta core.

From the 3D images, we can obtain information related to each phase in the sample; nonetheless, it is not possible to determine the bond created between the two layers nor the microstructure of each phase. In order to obtain this information, we used SEM images; the red squares in Figure 4a indicate the exact position at which the SEM images were taken, and the result is shown in Figure 7. The typical α–β lamellar phase can be observed in the Ti64 layer (Figure 7(1)). Small and rounded pores can also be detected, which indicates that sintering is in the last stage and confirms the assumption of low porosity (~5%). Three distinct phases are detected at the boundary between Ta and Ti64 particles. First, a stabilized β-Ti phase, obtained due to Ta diffusion into the crystalline net of Ti, is found at the interface because of the high concentration of Ta. This phase allows for the bonding between layers, as shown in Figure 7(2-1). Second, when moving away from the interface, Ta diffusion is reduced, and the martensitic Ti phase can be observed, as depicted in Figure 7(2-2). The martensitic phase is obtained during cooling as a result of an incomplete transition from the β → α phase, which was inhibited by the Ta atoms diffused into the crystalline net of Ti [42,43]. Lastly, the typical lamellar α-Ti and β-Ti phases of Ti64 alloy are found far from the interface (Figure 7(2-2)). This suggests that the junction between the layers is achieved by the diffusion of Ta into Ti. Furthermore, the microstructure of the core shows the shape of Ta particles, with small changes with respect to its initial conditions (Figure 7(3)). This indicates that the sintering of Ta is in the initial or intermediate stage, as intended for a reduced mechanical strength, which was expected because of the thermal cycle used for the sintering of the samples.

### 3.3. Permeability Analysis

**Figure 8:**
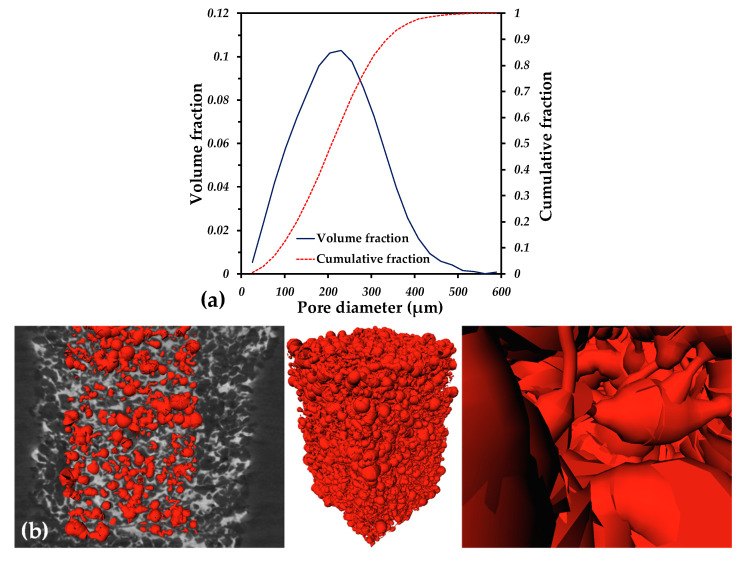
(**a**) Pore size distribution of the porous core; (**b**) 3D net of channels and pores in the core of the sample.

The permeability is related to the ability of fluid to pass throughout a porous media, and it is closely linked to the volume fraction, pore size, tortuosity, surface area and pore connectivity [44,45,46]. To determine the permeability, numerical simulations on the 3D microstructure issued from the tomography images were performed. Simulated flow lines are shown in Figure 6d, in which it is possible to note a low tortuosity. The permeability value is 6.06 × 10^−10^ m^2^, which was obtained after 20,000 iterations, as shown in Figure 6e. This value is in the same order of magnitude as the values reported for scaffolds fabricated by the space holder with a similar pore volume fraction (~55%) [47,48,49,50]. The difference between the permeability values is due to the pore size and is more important to the channel diameters and pore interconnectivity, as was suggested before [47,50]. The pore size distribution of the porous core is shown in Figure 8a. The pore size distribution ranges from 80 to 500 µm, and these values are similar to the ones of the pore formers’ size distribution. The statistical sizes of the pore size distribution are: 210 µm (d_50_), which is the median size, 90 µm (d_10_) and 350 µm (d_90_). With the aim of illustrating the complex shape of the porosity, a 3D net of channels and pores was built using the Skeleton module of the Avizo^®^ software; see Figure 8b. It demonstrates that fluid must pass through small channels connecting the larger pores. From the pore size distribution and Figure 8b, a channel size of d_10_ can be assumed, meaning 90 µm. Because of that, the permeability is low compared to that of the structures fabricated by additive manufacturing (AM) [49,51]. Nonetheless, the microstructure obtained by the space holder technique is mimicking the complexity of natural cancellous bone and represents an improvement compared to materials used nowadays for implants. The value of permeability is similar to the reported ones for different kinds of bones; for example, for trabecular bones, the permeability values range from 3 × 10^−11^ to 5 × 10^−10^ m^2^ for human proximal femurs and from 1 × 10^−8^ to 1 × 10^−9^ m^2^ for human vertebral bones [52]. In addition, the pore size distribution is in the size range recommended (100–400 µm) in order to promote the cell ingrowth and, with that, new bone that favors the osseointegration [53,54,55]. It was also reported that a pore size of 400 µm and a pore volume fraction higher than 60% improves the cell proliferation in vitro for Ti6Al4V scaffolds fabricated by AM [56]. The pore characteristics of the core are also in the reported range for good waste removal, cell seeding efficiency and vascularization, which plays a major role in new bone tissue ingrowth [57,58].

### 3.4. Mechanical Properties

**Figure 9:**
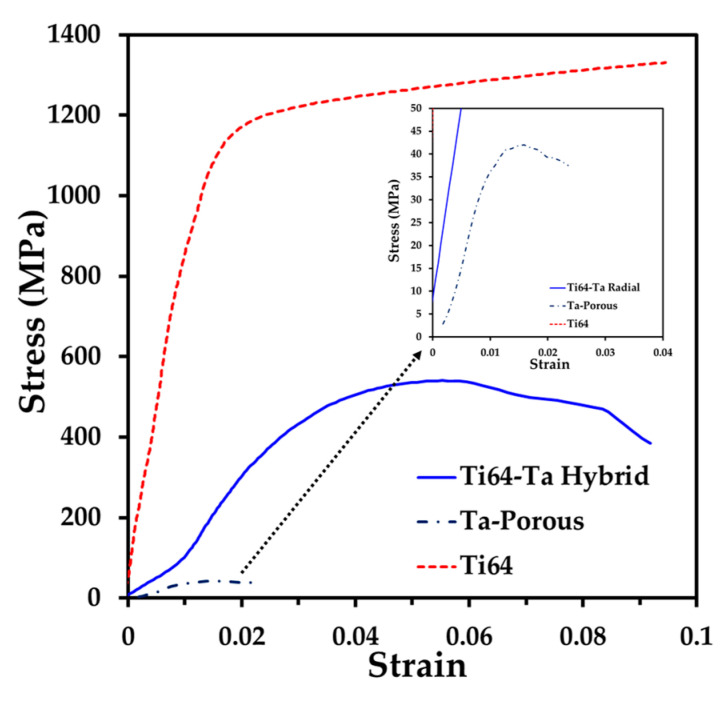
Stress–strain curves of simple compression tests of the bilayer sample and mono-material samples of porous Ta and Ti64, sintered under the same conditions.

The mechanical strength of the hybrid material sample was evaluated by compression tests. To have a better understanding, samples of Ti64 and porous Ta, with the same pore formers volume, were sintered under the same sintering cycle and tested under simple compression (Figure 9). The curve of Ti64 shows a linear increase in the stress as a function of the strain that corresponds to the elastic response. Next, the material reaches the yield, and during the plastic behavior, a continuous increment in the stress is noted as the strain increases. The ultimate strain was close to 10%. This behavior is similar to the one reported for Ti64 compacts sintered up to 1250 °C [59,60]. The behavior of the Ta porous sample is quite different, since after the elastic response, a maximum value of the stress is reached, and then the failure of the sample is obtained with a small strain (2.2%). This behavior is similar to the one reported for the compression or tension of porous Ta fabricated by the space holder technique [30,61]. The hybrid sample shows a lower slope in the elastic zone, and it also reaches a maximum value of the stress but with a larger strain than the Ta porous sample (9.2%) before the failure.

The Young’s modulus, E, and the yield strength, σ_y_, deduced from the stress–strain curves are listed in Table 1. The moduli of the Ti64 and porous Ta samples are 82.4 and 4 GPa, respectively. The value of Ti64 is according to the relative density reached at 92%, as reported elsewhere [13]. Meanwhile, the modulus of the porous Ta is similar to the one reported by Sukumar et al. [36], with the same pore volume fraction. The modulus of the hybrid sample is 16.3 GPa, i.e., under 20 GPa, which is the upper limit of the human bones [2]. This value is lower than the one estimated by the mixture’s rule, 43.2 GPa. This suggests that the porous core plays a major role in the stiffness of the hybrid material, and the bonding, developed at the interface by the diffusion of Ta, is strong. A similar finding was obtained when Ti6Al4V scaffolds with graded porosity were fabricated by selective laser melting (SLM). In this research by Liu et al., the authors fabricated scaffolds with various porosities and a gradient scaffold and reported the Young’s modulus values. When comparing the E values for all the scaffolds. we can observe that the scaffold with the lowest value of E is the one that drives the E value of the gradient scaffold, as in our case, where the porous core is the one that drives the E value for the two-layer sample [62].

The yield stress of the Ti64 and porous Ta is 867 MPa and 39.3 MPa, respectively. The yield stress of the hybrid material is 425 Pa, higher than the upper limit reported for human bones, 200 MPa [2]. This high value is due to the strengthening brought by the Ti64 shell layer that brings stability to the porous core. In addition, the so-called admissible strain (σ_y_/E) of the two-layer sample is 0.0289, which is much higher than the one measured for the Ti64 and porous Ta samples. This value is also higher than the one previously reported for porous Ta samples (around 0.0025) [36]. The value of the admissible strain is also higher than the one reported by Zaharin et al. [63] for the scaffold of Ti6Al4V fabricated by AM with cube or gyroid elements with similar pore volumes and pore sizes (0.0019). This is beneficial since it was reported that, for bone implant applications, high admissible strains are desired [64]. For example, the value for the cortical bone is about 0.02 [2], which is close to the one obtained for our hybrid material.

## 4. Conclusions

A novel hybrid material composed of a highly porous Ta core and a dense Ti64 shell was successfully fabricated, by pressing and sintering, for possible bone implant applications. The interface between the core and the shell exhibits a good bonding due to the atomic diffusion of Ta into Ti. 

The weight density of the sample was around 6 g/cm^3^, which is three times larger than the one of human bones but, in exchange, lower than the one of the materials used nowadays for bone implant applications, such as stainless steels and Co alloys. 

The pore size and volume fraction in the porous structure of the Ta core were controlled by the addition of pore formers; consequently, a permeability of 6 × 10^−10^ m^2^, which is close to the one of human bones, was obtained. In addition, the mechanical properties are also in the range of the ones reported for human bones, as we obtained a value of 16 GPa for E and 463 MPa for σ_y_, the porous layer being the one that dictates the mechanical strength. 

The presence of Ta is intended to accelerate the bone ingrowth, not just because of the good biocompatibility and osteoconductivity, but particularly because of the three-dimensional (3D) porous structure of the core. It was concluded that this hybrid material can improve the performance of bone implants due to the tailored characteristics and properties that might accelerate the osseointegration. An additional study of the material behavior in corrosive fluids and during cell and bone ingrowth is needed prior to using such a material for bone implant applications in the future.

## Figures and Tables

**Table 1:** Mechanical properties of Ti64, the porous sample and hybrid material.

Sample	E (Gpa)	σ_y_ (Mpa)	σ_y_/E (10^−3^)
Ti64	82.4 ± 1.2	867 ± 8.7	10.52
Porous Ta	4 ± 0.1	39.3 ± 0.8	9.82
Hybrid component	16 ± 0.4	463 ± 9.2	28.93

## Data Availability

The raw/processed data required to reproduce these findings cannot be shared at this time, as the data also form part of an ongoing study.
